# Self-management and self-efficacy of women with gestational diabetes mellitus: a systematic review

**DOI:** 10.1080/16549716.2022.2087298

**Published:** 2022-07-22

**Authors:** Savvato Karavasileiadou, Wafa Almegwely, Anwar Alanazi, Hanan Alyami, Sofia Chatzimichailidou

**Affiliations:** a Department of Community Health Nursing, College of Nursing, Princess Nourah bint Abdulrahman University, Riyadh, Saudi Arabia; b Medical - Surgical Nursing Department, College of Nursing, Princess Nourah bint Abdulrahman University, Riyadh, Saudi Arabia; c Department of Pathology, Hippokration General Teaching Hospital, Municipality of Thessaloniki, Greece

**Keywords:** Gestational diabetes mellitus, self-management, self-efficacy, glycemic control, systematic review, qualitative, diagnostic

## Abstract

**Background:**

Gestational diabetes mellitus (GDM) is a temporary form of diabetes induced by pregnancy and is potentially harmful to both the mother and fetus The impact of GDM diagnosis on pregnant women needs to be taken into account. This is related to the capacity for self-management of GDM, for which quality evidence is still lacking.

**Objective:**

to identify several aspects of self-management and self- efficacy for women with GDM.

**Method:**

Electronic databases were searched for studies related to the self-management, self-efficacy, and glycemic control of women with GDM, from January 2012 to January 2021. The extraction of study features was based on study location, reported research aims, study design, methodology, and the analytical approach, using Endnote Version X7.7.1. The Critical Appraisal Skills Program Qualitative Checklist (CASP) was used to assess quality, as recommended by the Cochrane Qualitative Research Methods Group.

**Results:**

Ten out of 70 studies were identified as meeting the established criteria and including a diverse population. The synthesis revealed seven major themes: preliminary psychological impact, communicating the diagnosis, knowledge of GDM, self-efficacy and self-management of GDM, risk perception, the burden of GDM, and gaining control. The benefits of a diagnosis were behavioral and were mostly crystalized if a particular level of self-management and self-efficacy was reached and women were able to have specific control over their diet and body weight. On the other hand, women reported that the diagnosis increased their responsibility, as they had to take extra precautions regarding their dietary regimen.

**Conclusion:**

Self-management and self-efficacy for GDM management are possible, despite the psychological hurdles that most women confront. There is still potential for improvement in terms of developing a healthy lifestyle that not only manages GDM for the best pregnancy result, but also prevents diabetes after pregnancy.

## Background

During the severe metabolic stress of pregnancy, gestational diabetes mellitus (GDM) occurs when the body is unable to maintain adequate glucose tolerance levels. A hormone released by the placenta stops the body from adequately utilizing insulin in this condition [1]. As a result, rather than being absorbed by the cells, glucose builds up in the blood. Unlike type 1 diabetes, gestational diabetes is not caused by a shortage of insulin [2], but by a variety of different hormones released during pregnancy. Insulin resistance is a condition that occurs when these hormones make insulin less effective. This is the reason why the symptoms disappear once the delivery has taken place. GDM is one of the most typical complications during pregnancy and is potentially harmful to the mother and the fetus as well [3]. According to the most recent estimates from the International Diabetes Federation (IDF), GDM affects over 14% of pregnancies globally, resulting in approximately 18 million newborns each year [4]. Cases of GDM are increasing globally. There are several reasons for this, such as obesity, advanced maternal age, and migratory problems [5].

The pathophysiologic basis of GDM can be summarized as a relevant insulin insufficiency. The placenta is in charge of providing water and nutrition to the fetus, as well as producing a variety of hormones to keep the pregnancy going [6]. The contra-insulin effect occurs when some hormones, such as cortisol, human placental lactogen, and estrogen, have a blocking effect on insulin, which normally begins around 20 to 24 weeks into the pregnancy [7]. The hormones generated by the placenta increase in quantity as the baby grows, increasing the risk of insulin resistance. In most cases, the pancreas can produce incremental insulin in response to insulin resistance [8]. Placental hormones are counter-regulatory to insulin thereby reducing its efficacy to maintain normal blood glucose [9]. Despite the fact that a number of research have looked into the effects of a GDM diagnosis, there hasn’t been a systematic review on self-efficacy, self-management, and glycemic control in women. The findings could help healthcare providers to better understand women’s attitudes and the repercussions of a diagnosis, revealing chances for assistance and advice. As a result, the aim of this systematic review is to identify features of self-management and self- efficacy in women with GDM.

## Methods

The Preferred Reporting Items for Systematic Reviews and Meta-Analyses (PRISMA) standards were followed for this study. It was determined to include primary research that had been published in peer-reviewed journals and had the following characteristics: pregnant women with a current diagnosis or a history of GDM were included. Collected qualitative data on the psychosocial effects of a GDM diagnosis on women at any stage of pregnancy and in the postpartum period, in which participants described their background or perspective on living with GDM It informed women about self-management and self-efficacy while they were undergoing diagnosis. There were particular limitations regarding countries, languages, and years the research may be published in. Furthermore, studies that focused on components of GDM other than self-management and self-efficacy were omitted; studies that dealt with women who had been diagnosed with diabetes prior to pregnancy were also omitted. Abstracts, letters, editorials, and opinions were not considered in the study.

### Methods for locating research in the database

To create a search strategy, a chosen mix of Medical Subject Headings phrases focused on three main areas: I) gestational diabetes mellitus, II) self-management and self-efficacy for gestational diabetes mellitus, and III) patient experience. Starting in January 2012 and ending in January 2021, the Google Scholar database was searched. The listed studies were subjected to a forward and backward citation search.

### Process of selection

Using Endnote Version X7.7.1, the lead investigator reviewed the titles and abstracts of retrieved references. The lead investigator independently reviewed all potentially qualifying full-texts. The abstracts were screened for discrepancies and potential eligibility that were then solved by consensus involving another reviewer. Considering the inclusion criteria, the full text of all the studies was revised in case of a discrepancy.

**Figure 1.**
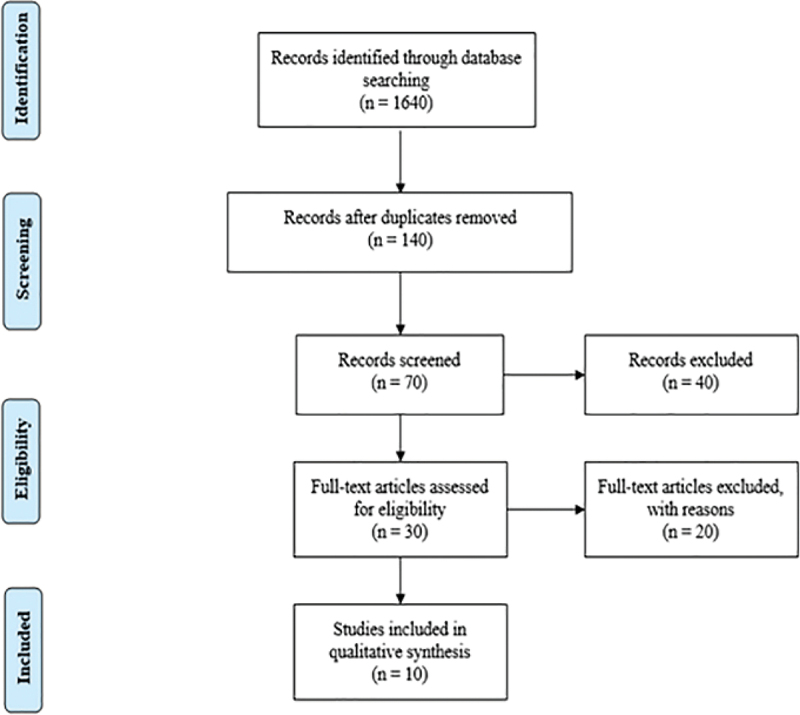
Search strategy.

In total, during the research, 1,640 studies were identified. After weeding out duplication and screening titles and abstracts, a total of 140 studies were assessed. A total of 70 studies were also identified through citation searching. The information was then taken from the ten studies that matched the review’s qualifying criteria. The search strategy is shown in Figure 1.

### Extraction of data

The principal investigator extracted study information such as study location, research aims, study design, methodology, and analytical strategy. Information on the diagnostic criteria for evaluating GDM in women was also gathered.

### Data synthesis

It was decided to apply a thematic analysis, as described by Thomas and Harden, to synthesise the study findings. One of the benefits for using thematic analysis is that it allows researchers to draw conclusions based on common themes in otherwise disparate studies and provide findings that directly advise health practitioners. Furthermore, the coding was inductive, meaning that the codes were derived from the data. To begin, relevant content about perceptions and experiences was retrieved and classified line by line. The lead investigator coded a selection of studies (n = 6) independently to build a coding methodology, and any discrepancies were resolved through discussion. The lead investigator classified an additional subset (n = 4) and noted Kappa = 0.87 inter-rater reliability. The chief investigator then applied the coding methodology to the remaining research, iteratively developing new codes as new concepts arose. The lead investigator then examined the links between the codes in order to develop descriptive themes across all of the trials. Moreover, related ideas were grouped together to form themes, and the less common requirements were divided into subthemes. Analytical themes were created during the last stages of synthesis and analysis. This was done to go beyond the primary investigations so that the results might be combined and accurately evaluated.

### Quality assessment

The Critical Appraisal Skills Programme Qualitative Checklist (CASP) was used to assess the quality. The Cochrane Qualitative Research Methods Group approves this method; hence it was chosen. Another reason is that it employs a systematic technique to evaluate three main areas: external validity assessment, methodological quality evaluation, and study validity. All critical appraisals were carried out by the lead investigator.

## Results

### Characteristics of the research

The studies as shown in Table 1 that were chosen used a wide range of data collection techniques. Out of ten studies, five were qualitative [10–15], one was based on a survey [16], and three were experimental [17–19].

**Table 1.** Study characteristics.

#	Author, Date & Country	Aim of Study	Method of data collection	Participants
1	Parsons et al. [10] 2018, UK	The aim of this research was to explore the experiences of GDM and GDM care for a group of women attending a large diabetes pregnancy unit in southeast London, UK, in order to improve care.	Qualitative study	Framework analysis was used to support an integrated analysis of data from six focus groups with 35 women and semi-structured interviews with 15 women, held in 2015. Participants were purposively sampled
2	Carolan et al. [11] 2013, Negara:Australia	Objective: to explore women’s experiences of self-managing their gestational diabetes.	Qualitative study	Data was collected using semi-structured interviews and one focus group. Participants included 15 women with a diagnosis of gestational diabetes who had experienced self-management of their condition.
3	Carolan et al. [12] 2018, Australia	Aimed to explore the factors that support and inhibit selfmanagement in pregnant women with gestational diabetes	Qualitative study	Semi structured interview and focus group discussion. 15 pregnant women with DMG with 28–38 weeks’ gestation were included
4	Neufeld et al. [13] 2011, Canada	To describe how Aboriginal women in an urban setting perceive dietary treatment recommendations associated with gestational diabetes mellitus (GDM).	Qualitative study	29 Aboriginal women who were diagnosed with DMG for a period of time in the last 5 years in Winnipeg, Manitoba Semi-structured explanatory model interview
5	Khooshehchin et al. [14] 2016, Iran	Study is aimed at a deeper understanding of women’s experiences of gestational diabetes and their perceived needs to inform future lifestyle interventions.	Qualitative study	Participants consisted of 12 pregnant women diagnosed with gestational diabetes in 24–36th week of pregnancyIn-depth interviews were conducted with participants, using semi-structured questions. Data saturation was obtained after 12 interviews.
6	Wah et al. [15] 2019	To explore the understanding and self-management experiences of Gestational diabetes among Chinese migrants.	Qualitative study	Data were collected through individual semi-structured face-to-face interviews. 18 pregnant women with DMG who are ethnic Chinese migrants living in Australia
7	Schmittdiel et al. [16] 2013, USA	Evaluation of a telephonic health-coaching service for patients at high risk of diabetes, as well as postpartum glucose test and a diabetes preventive educational program.	Survey	From January 1 to 23 August 2011, patients who engaged in wellness coaching. N = 1,427
8	Kim et al. [17] 2013, South Korea	The aim of this study was to see how an integrated self-management program affected self-management, glycemic control, and maternal identity in women with gestational diabetes (GDM).	Experimental	Women with GDM were recruited from Cheil General Hospital in Seoul, South Korea, and divided into experimental (n = 28) and control (n = 27) groups. N = 55
9	Jeon et al. [18] 2018, South Korea	The aim of this study was to see how a postnatal care program affected women with gestational diabetes mellitus’ self-efficacy, self-management, and glycemic control (GDM).	Experimental	Women with GDM were enrolled and randomly allocated to one of two groups: experimental (n = 30) or control (n = 32). The experimental group received a postnatal care program for women with GDM as an intervention. N = 62
10	Al-Hashmi et al. [19] 2019, Oman	The aim of this study was to see how successful a self-efficacy-enhancing intervention (SEEI) was at improving perceived self-efficacy and actual adherence to healthy behaviors in women with gestational diabetes (GDM).	Experimental	N = 90 adult Omani women with GDM were randomly assigned to one of two groups: a control group receiving conventional prenatal care or a SEEI group receiving SEEI.

### Quality appraisal

Two individual reviewers verified study papers for each article following PEDro scale quality presence or absence. It was assessed that most of the studies were of high quality, and the aims were clearly stated. The qualitative approaches utilized in all of the studies were appropriate since they attempted to explore or gather knowledge, opinions, or attitudes about GDM. On the other hand, studies that employed various designs and had lack of detail about methodology and other study techniques were ignored. Furthermore, data was collected in all of the investigations in order to effectively address the research question.

### Review analysis

The following are the seven themes that emerged from the data synthesis. 1) The psychological impact of the diagnosis, 2) communicating the diagnosis, 3) knowledge of GDM, 4) self-efficacy and GDM management, 5) risk perception, 6) GDM burden, and 7) obtaining control.

### Psychological impact

When women were first diagnosed with GDM, they reported experiencing failure, despair, self-blame, fear, perplexity, and concern, among other emotions. It was revealed that these women often concentrated on the ambiguity around the diagnostic prognosis, and that some even saw the condition as a life-changing experience. Some stated that they were unsure how to go on with the disease. It was common for women to feel an overwhelming sense of guilt and vulnerability. However, in some cases, the diagnosis was viewed favorably as an opportunity to make changes in one’s lifestyle. Some women said the diagnosis was a ‘wake-up call’ for them, and that they were grateful since they could now intervene and avert possibly harmful effects for themselves and their babies. Prior studies have reported similar results; for instance, Craig et al. [20] affirmed that a diagnosis of GDM has far-reaching implications that affect a wide range of women. The present biological approach utilized in the management of GDM is excessively fetal-centric and fails to recognize crucial psychological variables that contribute to women’s overall welfare and experience of pregnancy, according to Muhawva et al. [21].

### Communicating the diagnosis

Communication with healthcare providers and their families was also a prevalent feature in the findings of the studies examined. However, contact with healthcare professionals was mixed; some women stated that their interaction with their healthcare professionals was positive, while others noted that it was negative because of an unsupportive consultation and use of technical language. The primary issues that the women faced were limited availability of time to spend with healthcare professionals and a lack of understanding of healthcare professionals’ roles. Furthermore, the women also reported that the quality and level of information provided were contradictory, insufficient, or confusing, which was especially true regarding dietary changes. Some women even developed a dependency concerning monitoring, follow-ups, and general actions on how to deal with the disease. Some women stated that they had no choice of treatment, which resulted in feelings of fear and frustration. Another issue was the limited availability of information that prompted the women to seek information from other sources such as the internet.

There is no gold standard in terms of communication methods and public awareness campaigns, according to Breuing et al. [22], thus it is vital to concentrate on this area and develop novel approaches to avoid feeling angry about communication. When communication is robust, women will be better able to handle GDM independently.

### Knowledge of GDM

It was found that women had diverse levels of insight and information about GDM at various stages. Women who could explain the causes of GDM were able to accept and digest the diagnosis significantly faster than those who had no prior experience. Some of the women expressed their desire to have been better prepared for the diagnosis. Some women revealed issues with their learning curve concerning the dietary regimen. A majority of the women also stated that they faced challenges with adopting new habits so that GDM could be managed. This was difficult for these women because it required a trial-and-error approach. A general misunderstanding concerning the impact of GDM was also observed as some of the women thought GDM would transfer to their babies through breastfeeding.

### Self-efficacy & management of GDM

Pregnancy complications due to GDM can be reduced through stringent glycemic control and self-monitoring of glucose level. During gestation, insulin resistance is mainly observed in the skeletal muscles; therefore, aerobic exercise is highly recommended as a supportive and safe treatment plan to improve glucose metabolism through stimulation of insulin-dependent glucose uptake in both insulin-dependent and type 2 diabetes (T2DM) [15]. This mechanism involves fat-free mass availability to serve as a principal site for the disposal of insulin-dependent glucose. A recommended exercise plan before meal consumption in GDM patients improves metabolism and glucose effectiveness. Resistance exercise is another approach in GDM patients that involves voluntary skeletal muscle contraction against external resistance; however, the significance of this technique in attaining glycemic controls in the scientific literature is still occasional [23]. Another study by de Barros et al. [24] reported a significantly reduced insulin requirement in patients observed with resistance exercise compared to the control group by improving capillary glycemic control (P = 0.005).

Maternal and neonatal health can be improved through lifestyle interventions (diet and physical activity). Another approach [13,17,19] is to enhance awareness through a health counseling plan for a healthy lifestyle that includes diet, exercise, oral glucose tolerance test awareness, a ‘standard glucose tolerance test’ for earlier diabetes detection, self-monitoring, and weight loss via telephone to high-risk GDM patients, along with diabetes prevention education program and postpartum screening of glucose levels to prevent this chronic illness. GDM patients’ minimum goal is 5.8 mmol/l fasting capillary blood glucose levels and 7.8 mmol/l postprandial one-hour capillary blood glucose levels. However, if normal glycemic levels in GDM patients is not attainable, pharmacological intervention (oral hypoglycemic agents, insulin initiation) is required [25].

### Risk perception

Women’s risk perceptions before and after the diagnosis of GDM, as well as post-delivery were studied. The diagnosis astonished some women, especially those who were asymptomatic. However, there was disagreement on the health implications of the diagnosis some women perceived this (GDM) as having minimal long term consequences while others thought it was more hazardous. One of the most prominent concerns expressed by the women was the negative impact of GDM on their newborns. Another source of concern was the women’s diet, which they believed was too restrictive for the infant and would not offer all necessary nutrients. Some respondents were of the impression that the risk associated with GDM always disappeared after delivery.

‘Low perceived risk for T2DM might be a barrier to lifestyle change in women with recent GDM according to Chloe et al. [23]. Therefore, if the risk perception is managed effectively by healthcare professionals, this may possible lead to increased self-management and self-efficacy in women with GDM.

### Burden of GDM

The majority of the women said that being diagnosed with GDM added additional responsibilities and pressure to their daily lives. In their regular routines, treating and monitoring proved to be time-consuming. BGL measurements were time-consuming and exhausting, according to one common theme; there was a constant need to organize the means systematically and judiciously. Furthermore, the physical toll of GDM, such as side effects and therapy, was cited as a major concern. As a result, many women claimed that their family planning had been significantly impacted; some women even opted not to have children for a period of time because they were afraid of having a similar experience.

### Gaining control

One of the common words discovered during the analysis was ‘control.’ The majority of the women stated that initially, they experienced a lack of control of their emotions. However, once self-management and self-efficacy were reached, there was a specific level of control. Some women even stated that they had achieved self-empowerment as they gained control through self-management. Taking control entailed recognizing the changes that were unavoidable due to their lifestyle, self-care, and self-education.

## Discussion

This review of qualitative evidence on the experience of self-efficacy, self-management, and glycemic control in women with GDM emphasizes the psychological and social effects of GDM diagnosis. From the review, it was discovered that the benefits of diagnosis were by and large behavioral and mostly crystalized if a particular level of self-management and self-efficacy was reached and women were able to have a certain degree of control over their diets and body weight. However, women reported that the diagnosis increased their responsibility because they now had to take extra precautions concerning their dietary regimens. Other disadvantages included budgetary limits and cultural problems as a result of the required atypical eating habits.

The psychological effects on the women were severe, and they were frequently isolated for lengthy periods of time. In addition, the women included in the study reported that they were provided with conflicting information; this led to difficulties in obtaining adequate levels of self-management and self-efficacy. Another important conclusion of this study was the lack of knowledge about the risk of GDM, particularly the disease’s long-term implications. A lack of health literacy could be one factor contributing to a lack of understanding of GDM.

The findings of this study highlight some of the overlooked aspects and themes about a GDM diagnosis. They include a lack of individualized care, a lack of options regarding the critical elements of care such as giving birth, and a lack of options regarding follow-ups with healthcare professionals. Some of the women felt abandoned to a significant extent after the delivery had taken place. Researchers have previously stated that postnatal screening and persistent lifestyle changes to prevent future diabetes appear to fade after birth, owing to the lack of a driver to protect the unborn child [26].

Various forms of content can be informed by a synthesis of women’s experiences with GDM diagnosis. It can be used for communication before and after diagnosis. The findings also provide an opportunity for healthcare professionals to impart adequate knowledge to women so their self-management process may be dealt with more effectively. The study also highlights the need for service redesign, which can be accomplished by enhancing community-based care for women diagnosed with GDM. This would help with self-management and self-efficacy because of the burden associated with attending hospital appointments.

The two studies [15,16] from South Korea emphasized glycemic self-management during gestation and postnatal care through education and training programs for GDM patients for improved self-efficiency. Six studies from Southeast Asia and the Middle East [6, 11–13, 17, 18] also supported the establishment of health coaching plans for GDM women to ensure timely self-management of diabetes through food, exercise, and treatment regimen awareness, as well as self-blood sugar monitoring. This region has a solid requirement for initiating health policies, including diabetic educators’ involvement at the primary care level through electronic media to assure timely awareness in patients and to avoid the loss of follow-up. One of the studies conducted in Germany [14] predicted cardiovascular events in patients with diabetes mellitus and coronary artery calcification as a prognostic factor and emphasized the need to monitor diabetes in patients as a concurrent condition. A study from the United States (USA) [19] addressed gestational diabetes mellitus as a global health concern. It concluded that healthcare systems and policymakers should develop an infrastructure to prevent this chronic illness and decrease its incidence by improving efficient lifestyle approaches.

## Strengths & limitations

Studies with women of varied demographic characteristics and ethnic groups were included in this systematic review. The principal themes discovered were common to the majority of the research. External validity concerning the studies’ authenticity is provided by the high rates of involvement in the studies assessed. A robust technique to improve credibility involved comparing the coding between authors, discussing the results, and reaching a consensus. The majority of the studies were of good or satisfactory quality. Finally, despite the fact that the data came from a wide range of people, the study focused mostly on middle-income countries, which are thought to have more well-established and evidence-based healthcare systems compared with low-income countries.

Women’s income and/or education levels are linked to GDM management. This is significant because educated women are more likely to be aware of the importance of lifestyle and self-management; in addition, women with higher incomes may be able to afford a better diet and medical care. Carolan et al. findings are consistent with a higher usage of insulin in the clinic, where worse dietary adherence and higher rates of hyperglycemia are assumed to be a result of less maternal education and comprehension. Whatever the causes, the research participants’ rates of dietary self-management were much lower than the suggested 65–90% of women. This trait might possibly be a result of the lack of culturally relevant educational opportunities for women in this area [12].

## Future research

According to Sumali Hewage’s qualitative results, working women reported facing obstacles such as a lack of blood glucose self-monitoring, difficulty with food control, and a lack of time for prescribed exercise [27]. Despite the fact that physical exercise has been found to help women with GDM control their blood glucose levels, pregnant women in Singapore are less likely to be active, particularly in the latter stages of pregnancy [28]. Other obligations, such as work-related activities, may exacerbate this lack of behavioral change. Gaps in GDM treatment have been identified, highlighting the need for appropriate interventions that can fit into hectic schedules [29].

Globally, health information technology and health-care-related technologies have advanced at a breakneck pace [30]. The rise of technological tools like smartphones and digitally enabled therapies as a result has sparked a lot of interest in their application in public health and lifestyle medicine, both in research and practice [31]. Digital technologies can be integrated into innovative techniques for lifestyle modification relevant to population health [32,33] due to their cost effectiveness and promising ability to boost health behavior change [34]. The primary purpose of digital health is to encourage healthy lifestyle choices for effective disease prevention and management [32]. Virtual consultation, online support groups, and web-based professional counselling on health concerns have been proven to have a good influence on disease prevention using online and smartphone health apps [34]. Smartphone and other Internet-based health platforms promote greater patient involvement, appointment keeping, and early reporting of any health indicators of concern to health care practitioners, in addition to minimizing needless visits to health care services for health advice [33]. As a result, public interest in more inexpensive and simpler access to their health requirements is growing [35].

The findings of this analysis show that there is still opportunity for more research into the psychological advantages and risks of GDM. In addition, because some of the women said that being diagnosed with GDM meant taking on more duties, a novel model for improving self-management and self-efficacy with GDM may be examined and developed.

## Conclusion

The consequences of a GDM diagnosis are extensive and multifaceted. Despite the psychological challenges that most women confront, self-management and self-efficacy in the context of GDM can be improved. After pregnancy, there is a chance to develop a healthy lifestyle and avoid the onset of T2DM. This goal could be reached by addressing the probable negative consequences of a GDM diagnosis, such as social isolation and poor psychological repercussions.

## Paper context

Evidence on self-management of GDM, a common condition due to increased prevalence of pregnancies and obesity, is lacking. Our study showed that despite the challenges and the increased sense of responsibility following the diagnosis, with regards to food control and physical exercise, self-management and self-efficacy through behavioral and lifestyle modification is feasible. This could be further improved by use of mobile applications, whereas online support groups could address possible psychologic repercussion and social isolation.

## Appendix.

**PEDro Scale**:

**Table:**

S.No	Specifications	No	Yes	Where
1	Eligibility criteria were specified.			
2	Subjects were randomly allocated to groups.			
3	Allocation was concealed.			
4	The groups were similar at baseline regarding most critical prognostic indicators.			
5	There was blinding of all subjects.			
6	There was blinding of all therapists.			
7	There was blinding of all assessors who measured at least one key point.			
8	The measure of at least one key outcome was obtained from more than 85% of the subjects initially allocated to groups.			
9	All subjects for whom outcome measures were available received the treatment or control condition as allocated or, where this was not the case, data for at least one key outcome was analyzed by ‘intention to treat’.			
10	The results of between-group statistical comparisons are reported for at least one key outcome.			
11	The study provides both point measures and measures of variability for at least key outcomes.			
